# A multimarker panel for predicting short-term outcome after intravenous thrombolysis in acute ischemic stroke: a retrospective study

**DOI:** 10.3389/fmed.2026.1913156

**Published:** 2026-08-10

**Authors:** Peng Jiang, Baoli Xu, Zhouquan Hu, Yong Tang, Xiaojia Luo, Bhushan Sandeep

**Affiliations:** 1 Department of Emergency, West China School of Medicine, Sichuan University, Sichuan University affiliated Chengdu Second People’s Hospital, Chengdu Second People’s Hospital, Chengdu, Sichuan, China; 2 Department of Cardio-Thoracic Surgery, West China School of Medicine, Sichuan University, Sichuan University affiliated Chengdu Second People’s Hospital, Chengdu Second People’s Hospital, Chengdu, Sichuan, China

**Keywords:** acute ischemic stroke, biomarkers, intravenous thrombolysis, NIHSS score, nomogram, prognosis, risk stratification, systemic immune-inflammation index

## Abstract

**Objective:**

To evaluate the predictive efficacy of a multimarker panel combining the National Institutes of Health Stroke Scale (NIHSS) score immediately after intravenous thrombolysis (IVT) with the Systemic Immune-Inflammation Index (SII), baseline blood glucose (Glu), and plasma uric acid (UA) for short-term neurological prognosis in patients with acute ischemic stroke (AIS).

**Methods:**

This single-center retrospective cohort study enrolled 132 consecutive AIS patients who received IVT at Chengdu Second People’s Hospital between January 2021 and December 2021. Clinical demographics, laboratory parameters, and NIHSS scores at admission (NIHSS1) and immediately after thrombolysis (NIHSS2) were collected. The modified Rankin Scale (mRS) at 90 days was used to classify patients into good prognosis (mRS 0-2) and poor prognosis (mRS 3–6) groups. Univariate and multivariate logistic regression analyses were performed to identify independent risk factors for poor prognosis. Receiver operating characteristic (ROC) curve analysis, calibration assessment, and decision curve analysis were conducted to evaluate and compare the predictive performance of individual and combined markers. Subgroup and sensitivity analyses were performed to validate model robustness.

**Results:**

Among the 132 enrolled patients, 55 (41.67%) had poor prognosis at 90 days. Multivariate logistic regression analysis identified NIHSS2 score (OR = 1.317, 95%CI: 1.092–1.588, *P* = 0.004), baseline Glu (OR = 1.389, 95%CI: 1.083–1.781, *P* = 0.010), UA (OR = 1.008, 95%CI: 1.001–1.014, *P* = 0.028), and SII (OR = 1.001, 95%CI: 1.000–1.002, *P* = 0.039) as independent risk factors for poor prognosis. The combined model incorporating these four indicators demonstrated superior predictive performance with an area under the curve (AUC) of 0.928 (95%CI: 0.870–0.966, *P* < 0.001), sensitivity of 98.18%, specificity of 72.73%, and Youden index of 0.709. The combined model significantly outperformed individual predictors: UA (*Z* = 5.276, *P* < 0.001), SII (*Z* = 4.031, *P* < 0.001), baseline Glu (*Z* = 3.595, *P* < 0.001), and NIHSS2 alone (*Z* = 2.124, *P* = 0.034). Calibration assessment demonstrated good fit (Hosmer-Lemeshow χ^2^ = 6.847, *P* = 0.553), and decision curve analysis confirmed net clinical benefit across threshold probabilities of 10–80%. Subgroup analyses demonstrated consistent excellent performance across age, sex, stroke severity, and thrombolytic agent subgroups (AUC range: 0.91–0.95). Leave-one-out sensitivity analysis confirmed that each biomarker contributed unique prognostic information to the combined model.

**Conclusion:**

The multimarker panel combining post-thrombolysis NIHSS score, SII, baseline Glu, and UA provides a simple, readily available, and highly effective approach for predicting short-term neurological prognosis in AIS patients after IVT. The combined assessment significantly improves predictive accuracy compared to individual markers alone, supporting its potential clinical utility for early risk stratification and individualized treatment decision-making. However, these findings require prospective multicenter validation before routine clinical implementation can be recommended.

## 1 Introduction

Acute ischemic stroke (AIS) remains one of the leading causes of mortality and long-term disability worldwide, imposing substantial socioeconomic burdens on healthcare systems and affected families (1). According to the Global Burden of Disease Study 2019, stroke is the second leading cause of death globally, with ischemic stroke accounting for approximately 62.4% of all stroke incidents (2). The disability-adjusted life years (DALYs) attributable to stroke have increased significantly over the past three decades, highlighting the urgent need for improved therapeutic strategies and prognostic assessment tools (3).

Intravenous thrombolysis (IVT) with recombinant tissue plasminogen activator (rt-PA) remains the cornerstone of early reperfusion therapy for eligible AIS patients, with established efficacy in reducing neurological disability when administered within the therapeutic time window (4, 5). Current international guidelines, including the 2019 update from the American Heart Association/American Stroke Association (AHA/ASA) and the 2018 Chinese guidelines, recommend IVT as the first-line treatment for AIS patients presenting within 4.5 h of symptom onset, with selected patients potentially benefiting from extended window protocols (6, 7). However, despite timely thrombolytic therapy, approximately 30–50% of patients fail to achieve favorable functional outcomes, and 6–10% experience symptomatic intracerebral hemorrhage (sICH), a devastating complication associated with high mortality (8, 9).

The heterogeneity in treatment response and post-thrombolysis outcomes underscores the critical importance of early and accurate prognostic assessment. Reliable prediction of neurological prognosis enables clinicians to communicate realistic expectations to patients and families, optimize post-thrombolysis management strategies, identify candidates for adjunctive therapies such as mechanical thrombectomy or neuroprotective interventions, and allocate healthcare resources appropriately (10). Furthermore, early recognition of high-risk patients facilitates intensified monitoring, aggressive blood pressure management, and timely intervention for complications, potentially improving overall outcomes (11).

The pathophysiology of ischemic stroke involves complex interactions between vascular occlusion, energy failure, excitotoxicity, oxidative stress, neuroinflammation, and blood-brain barrier (BBB) disruption (12). Emerging evidence suggests that systemic inflammatory responses, metabolic disturbances, and oxidative stress markers play pivotal roles in determining stroke severity and treatment outcomes (13). The Systemic Immune-Inflammation Index (SII), calculated as platelet count × neutrophil count/lymphocyte count, represents a novel composite biomarker that integrates thrombotic, innate immune, and adaptive immune pathways, offering a comprehensive assessment of the systemic inflammatory state (14, 15). Recent meta-analyses have demonstrated that elevated SII is significantly associated with increased mortality (OR = 1.58, 95%CI: 1.23–2.02) and poor functional outcomes (OR = 2.03, 95%CI: 1.63–2.52) in stroke patients (16).

Hyperglycemia, whether reflecting stress responses or underlying diabetes mellitus, exacerbates ischemic brain injury through multiple mechanisms including enhanced oxidative stress, mitochondrial dysfunction, augmented platelet activation, and impaired fibrinolysis (17, 18). The Stress Hyperglycemia Ratio (SHR) and baseline glucose levels have been consistently associated with poor outcomes after thrombolysis, with elevated admission glucose predicting increased sICH risk and reduced odds of functional independence (19, 20).

Uric acid (UA) exhibits a complex dual role in ischemic stroke pathophysiology. On one hand, UA possesses antioxidant properties, scavenging free radicals and protecting against oxidative stress-induced neuronal damage (21). Conversely, hyperuricemia promotes endothelial dysfunction, vascular smooth muscle proliferation, low-density lipoprotein oxidation, and systemic inflammation, potentially worsening stroke outcomes (22, 23). The prognostic significance of UA in AIS patients undergoing reperfusion therapy remains controversial, with studies reporting conflicting results regarding its neuroprotective versus detrimental effects (24, 25).

The National Institutes of Health Stroke Scale (NIHSS) score serves as the gold standard for quantifying neurological deficit severity in AIS and has been validated as a robust predictor of short-term and long-term outcomes (26). While admission NIHSS (NIHSS1) provides baseline severity assessment, the NIHSS score immediately after thrombolysis (NIHSS2) may better reflect treatment response and early neurological changes, potentially offering superior prognostic information (27). However, relying solely on clinical scales has limitations, as they may not capture underlying biological processes driving secondary injury and recovery (28).

Several multimarker prognostic models have been proposed for stroke outcome prediction. The ASTRAL score incorporates age, stroke severity, time from symptom onset, glucose level, and visual fields to predict functional outcomes, achieving an AUC of approximately 0.76 (29). The iScore integrates age, sex, stroke severity, and comorbidities with comparable performance (30). More recently, the SICFAIL, STRAWINSKI, and PREDICT studies demonstrated that integrating blood-based biomarkers (copeptin, NT-proBNP, MR-proANP) into established clinical scores modestly improved discrimination and calibration for functional outcome prediction after AIS, with AUC values ranging from 0.76 to 0.80 (28). However, these biomarker-enhanced models rely on specialized laboratory assays that may not be readily available in all clinical settings. Furthermore, the CoRisk score, incorporating copeptin, age, NIHSS, and recanalization therapy, achieved an AUC of 0.819 but requires copeptin measurement (31). Machine learning-based approaches have reported AUC values of 0.85–0.90, though their clinical interpretability and generalizability remain concerns (32). Despite these advances, existing models either require specialized biomarkers, complex computations, or have achieved only modest discriminative performance, highlighting the need for simpler, readily available multimarker approaches that can be implemented across diverse healthcare settings. Given the multifactorial nature of stroke prognosis and the complementary information provided by clinical, inflammatory, and metabolic markers, we hypothesized that a multimarker approach combining post-thrombolysis NIHSS with readily available laboratory parameters (SII, baseline Glu, and UA) would enhance predictive accuracy for short-term neurological outcomes in AIS patients after IVT. This study aimed to develop and validate such a combined prognostic model, comparing its performance against individual predictors and assessing its potential clinical utility for early risk stratification.

## 2 Materials and methods

### 2.1 Study design and setting

This was a single-center retrospective cohort study conducted at the Department of Emergency Medicine, Chengdu Second People’s Hospital, a comprehensive tertiary care hospital in Chengdu, China. The study was approved by the Institutional Ethics Committee of Chengdu Second People’s Hospital [approval number: (KY)PJ2026269], and the requirement for informed consent was waived due to the retrospective nature of the analysis. All procedures were performed in accordance with the ethical standards of the Declaration of Helsinki.

### 2.2 Study population

Consecutive AIS patients who received IVT at our institution between January 2021 and December 2021 were retrospectively screened for eligibility. The diagnosis of AIS was confirmed by experienced neurologists based on clinical presentation, neurological examination, and neuroimaging findings (computed tomography or magnetic resonance imaging) according to the Chinese Guidelines for Diagnosis and Treatment of Acute Ischemic Stroke 2018 (7).

Inclusion criteria were: (1) Age ≥ 18 years; (2) Clinical diagnosis of AIS with symptom onset within 6 h prior to treatment; (3) Receipt of intravenous thrombolysis with either alteplase (for patients presenting within 4.5 h) or urokinase (for patients presenting between 4.5 and 6.0 h), administered according to current guideline recommendations; (4) Availability of complete baseline clinical data, laboratory results, and NIHSS scores; (5) Completion of 90-day follow-up for functional outcome assessment.

Exclusion criteria were: (1) Severe hepatic dysfunction (Child-Pugh Class C or bilirubin > 3 times upper limit of normal); (2) Severe renal dysfunction (estimated glomerular filtration rate < 30 mL/min/1.73 m^2^ or requiring renal replacement therapy); (3) Active autoimmune diseases or immunosuppressive therapy; (4) Hematological disorders affecting blood cell counts (e.g., leukemia, myelodysplastic syndrome); (5) Malignancy with expected survival < 6 months; (6) Severe pre-existing neurological deficits with modified Rankin Scale (mRS) score ≥ 3 prior to the index stroke; (7) Missing critical data precluding analysis.

#### 2.2.1 Sample size consideration

**FIGURE 1:**
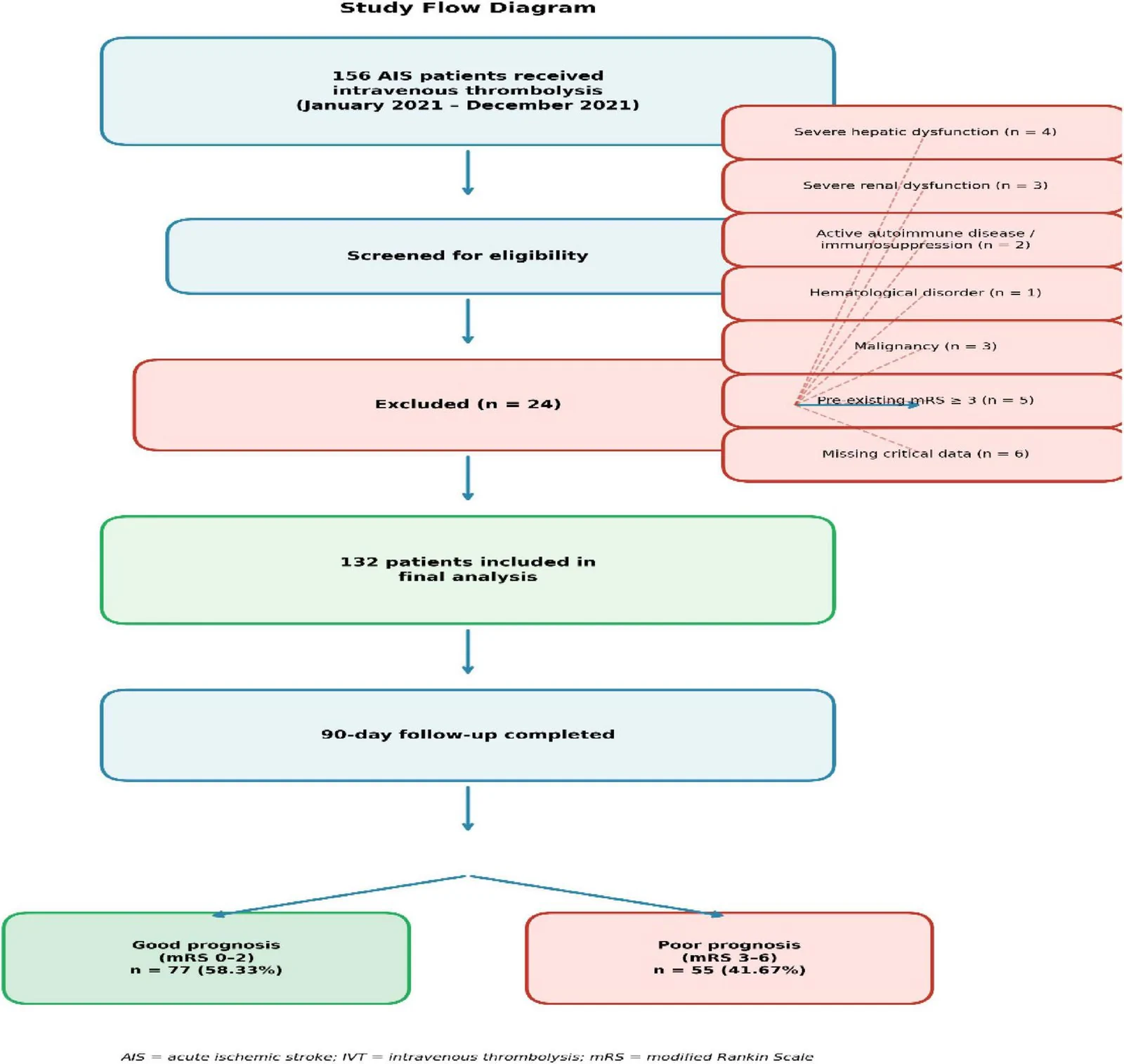
Study flow diagram. CONSORT-style flow diagram illustrating patient screening, inclusion, and exclusion processes. Of 156 patients who received intravenous thrombolysis during the study period, 132 were included in the final analysis after applying exclusion criteria.

To ensure adequate statistical power and minimize the risk of model overfitting, we conducted a sample size assessment prior to analysis. According to established guidelines for prognostic model development, a minimum of 10 events per variable (EPV) is recommended for multivariable logistic regression to achieve stable parameter estimates (33). In our cohort, 55 patients experienced poor outcomes (events), and the final multivariate model included 4 independent predictors, yielding an EPV ratio of 13.75:1 (55 events / 4 variables). This exceeds the minimum recommended threshold and approaches the more conservative standard of 20 events per variable suggested by some methodologists for reliable model development (34). Additionally, we assessed multicollinearity using variance inflation factors (all VIF < 3.0) and performed bootstrap internal validation (1,000 resamples) to evaluate model stability. The consistency of our findings across subgroup and sensitivity analyses further supports the statistical robustness of the model despite the modest sample size. Nevertheless, we acknowledge that larger derivation cohorts would further reduce the risk of overfitting and improve precision of effect estimates. Figure 1 shows the Study Flow Diagram. CONSORT-style flow diagram illustrating patient screening, inclusion, and exclusion processes. Of 156 patients who received intravenous thrombolysis during the study period, 132 were included in the final analysis after applying exclusion criteria.

### 2.3 Data collection and variables

Clinical data were extracted from the hospital’s electronic medical record system and stroke registry database by trained research personnel using a standardized case report form. The following variables were collected:

Demographic and clinical characteristics: Age, sex, body mass index (BMI), medical history (hypertension, diabetes mellitus, atrial fibrillation, prior stroke, coronary artery disease, smoking status), pre-stroke mRS score, onset-to-needle time (ONT), onset-to-door time, blood pressure parameters (systolic and diastolic blood pressure at admission and post-thrombolysis), and stroke subtype classification (TOAST criteria).

Laboratory parameters: All laboratory tests were performed on venous blood samples obtained upon emergency department arrival, prior to thrombolysis initiation. Complete blood count parameters including white blood cell count (WBC), neutrophil count (Neu), lymphocyte count (Lym), monocyte count, platelet count (PLT), hemoglobin (Hb), and hematocrit were measured using automated hematology analyzers. Biochemical parameters included serum creatinine (Cr), blood urea nitrogen, uric acid (UA), baseline blood glucose (Glu), admission random glucose, glycated hemoglobin (HbA1c, when available), total cholesterol, triglycerides (TG), low-density lipoprotein cholesterol (LDL-C), high-density lipoprotein cholesterol, and coagulation parameters (prothrombin time, activated partial thromboplastin time, international normalized ratio, fibrinogen).

The Systemic Immune-Inflammation Index (SII) was calculated using the formula: SII = (Neu × PLT)/Lym, where Neu represents neutrophil count ( × 10^9^/L), PLT represents platelet count ( × 10^9^/L), and Lym represents lymphocyte count ( × 10^9^/L) (14).

Neurological assessment: NIHSS scores were assessed by certified neurologists at admission (NIHSS1) and immediately after completion of thrombolysis (NIHSS2), typically within 30–60 min post-infusion. All NIHSS assessors underwent standardized training and inter-rater reliability verification.

### 2.4 Treatment protocol

Intravenous thrombolysis was administered according to institutional protocols aligned with national and international guidelines (6, 7). Patients presenting within 4.5 h of symptom onset received intravenous alteplase at a dose of 0.9 mg/kg (maximum 90 mg), with 10% of the total dose given as a bolus over 1 min and the remainder infused over 60 min. Patients presenting between 4.5 and 6.0 h received intravenous urokinase at a dose of 1.0 million units, infused over 30 min. All patients were managed in the stroke unit or intensive care unit with continuous monitoring of vital signs, neurological status, and potential complications. Post-thrombolysis blood pressure was maintained below 180/105 mmHg for at least 24 h. Antiplatelet therapy was initiated 24 h after thrombolysis following confirmation of absence of hemorrhagic transformation on repeat imaging.

### 2.5 Outcome assessment

The primary outcome was short-term neurological functional prognosis at 90 days after stroke onset, assessed using the modified Rankin Scale (mRS) (35). The mRS is a validated 7-point ordinal scale ranging from 0 (no symptoms) to 6 (death), with scores of 0–2 indicating functional independence (good prognosis) and scores of 3–6 indicating significant disability or death (poor prognosis). Follow-up assessments were conducted through structured telephone interviews, outpatient clinic visits, or review of medical records by trained research nurses blinded to baseline biomarker values. For patients who could not be contacted directly, information was obtained from caregivers or family members using standardized questionnaires.

Secondary outcomes included in-hospital complications (sICH defined according to the European Cooperative Acute Stroke Study II criteria (36), stroke-associated pneumonia, deep vein thrombosis, pulmonary embolism), length of hospital stay, and all-cause mortality during hospitalization and at 90 days.

### 2.6 Statistical analysis

Statistical analyses were performed using SPSS version 21.0 (IBM Corp., Armonk, NY, United States) and R version 4.3.1 (R Foundation for Statistical Computing, Vienna, Austria). Continuous variables were tested for normality using the Shapiro-Wilk test and visual inspection of Q-Q plots. Normally distributed continuous variables were expressed as mean ± standard deviation (SD) and compared between groups using independent samples *t*-tests. Non-normally distributed continuous variables were expressed as median (interquartile range, IQR) and compared using the Mann-Whitney U test. Categorical variables were expressed as frequencies (percentages) and compared using Pearson’s chi-square test or Fisher’s exact test as appropriate.

Univariate logistic regression analysis was performed to identify potential risk factors associated with poor prognosis. Variables with *P* < 0.10 in univariate analysis were entered into multivariate logistic regression analysis using backward stepwise selection (likelihood ratio method) to identify independent predictors. The variance inflation factor (VIF) was calculated to assess multicollinearity among predictors, with VIF > 10 considered indicative of significant multicollinearity. Spearman correlation analysis was performed to evaluate intercorrelations among continuous variables.

Receiver operating characteristic (ROC) curve analysis was conducted to evaluate the predictive performance of individual biomarkers and the combined model. The area under the ROC curve (AUC) with 95% confidence interval (CI) was calculated. Sensitivity, specificity, positive predictive value (PPV), negative predictive value (NPV), and Youden index (sensitivity + specificity − 1) were determined at optimal cutoff points identified using the Youden index method. The DeLong test was used to compare AUC values between different models (37).

Calibration of the combined model was assessed using the Hosmer-Lemeshow goodness-of-fit test, with *P* > 0.05 indicating adequate calibration. Decision curve analysis (DCA) was performed to evaluate the net benefit of the combined model across different threshold probabilities, comparing it against treat-all and treat-none strategies (38).

Subgroup analyses were performed stratified by age (<75 vs. ≥ 75 years), sex, baseline stroke severity (NIHSS1 ≤ 7 vs. > 7), thrombolytic agent (alteplase vs. urokinase), and diabetes status. Leave-one-out sensitivity analysis was conducted by sequentially removing each of the four independent predictors from the combined model and recalculating the AUC to quantify the unique contribution of each biomarker. All statistical tests were two-tailed, and *P* < 0.05 was considered statistically significant. Missing data was handled using complete case analysis, as the proportion of missing data was < 5% for all variables.

## 3 Results

### 3.1 Baseline characteristics

A total of 156 AIS patients received IVT during the study period. After applying exclusion criteria, 132 patients were included in the final analysis. Among the enrolled patients, 81 (61.36%) were male and 51 (38.64%) were female, with a mean age of 70.90 ± 13.03 years (range: 38–94 years). The median onset-to-needle time was 158.5 min (IQR: 118–208 min). Alteplase was administered to 98 patients (74.24%) and urokinase to 34 patients (25.76%).

During hospitalization, 15 patients (11.36%) developed cerebral hemorrhage (9 symptomatic, 6 asymptomatic), 37 patients (28.03%) developed stroke-associated pneumonia, and 6 patients (4.55%) died. At 90-day follow-up, 77 patients (58.33%) achieved good prognosis (mRS 0–2) and 55 patients (41.67%) had poor prognosis (mRS 3–6), including 6 deaths.

Comparison of baseline characteristics between good prognosis and poor prognosis groups is summarized in Table 1. Patients with poor prognosis were significantly older (73.80 ± 13.56 vs. 68.83 ± 12.41 years, *P* = 0.031), had higher prevalence of atrial fibrillation (43.64% vs. 12.99%, *P* = 0.004), and longer onset-to-needle times (178 vs. 145 min, *P* = 0.040). No significant differences were observed in sex distribution, hypertension, diabetes mellitus, smoking history, or systolic blood pressure between the two groups.

**TABLE 1:** Comparison of baseline characteristics between good prognosis and poor prognosis groups.

Variable	Good prognosis group (*n* = 77)	Poor prognosis group (*n* = 55)	*t*/*Z*/χ^2^	*P*
Sex			0.205	0.718
Male (n, %)	46 (59.74)	35 (63.64)		
Female (n, %)	31 (40.26)	20 (36.36)		
Age (years)	68.83 ± 12.41	73.80 ± 13.56	2.182	0.031
History of hypertension (n, %)	49 (63.63)	33 (60.00)	0.042	0.887
History of diabetes (n, %)	20 (25.97)	9 (16.36)	1.122	0.402
History of AF (n, %)	10 (12.99)	24 (43.64)	9.067	0.004
Smoking history (n, %)	27 (35.06)	18 (32.72)	0.038	0.863
ONT (min)	145 (112.5, 193.5)	178 (129, 229)	–2.056	0.040
Systolic blood pressure (mmHg)	150.08 ± 21.96	154.58 ± 26.12	1.073	0.285
Neu (10^9^/L)	4.38 (3.13, 5.55)	5.40 (4.09, 7.42)	−3.088	0.002
Lym (10^9^/L)	1.80 (1.40, 2.70)	1.40 (1.10, 2.28)	−2.161	0.031
Hb (g/L)	143.12 ± 20.87	137.09 ± 20.99	−1.631	0.105
PLT (10^9^/L)	188.30 ± 64.23	177.20 ± 56.15	−1.030	0.305
TG (mmol/L)	1.07 (0.83, 1.60)	1.06 (0.76, 1.54)	−0.224	0.823
LDL (mmol/L)	2.34 (1.93, 3.21)	2.41 (1.18, 3.04)	−0.051	0.960
UA (μmol/L)	343.21 ± 106.72	414.07 ± 110.24	3.710	< 0.001
Cr (μmol/L)	69 (59.5, 87.5)	85 (69, 100)	−3.029	0.001
SII (10^9^/L)	488 (263, 798)	1107 (640, 1717)	−5.629	< 0.001
Admission Glu (mmol/L)	7.46 ± 3.05	7.80 ± 2.63	0.666	0.506
Baseline Glu (mmol/L)	5.82 ± 2.12	7.73 ± 2.41	4.827	< 0.001
NIHSS1 (score)	4 (2, 8)	15 (7, 18)	−6.613	< 0.001
NIHSS2 (score)	2 (1, 4)	14 (7, 17)	−7.518	< 0.001

Data are presented as mean ± SD, median (IQR), or n (%). ONT, onset-to-needle time; AF, atrial fibrillation; Neu, neutrophil count; Lym, lymphocyte count; Hb, hemoglobin; PLT, platelet count; TG, triglycerides; LDL, low-density lipoprotein; UA, uric acid; Cr, creatinine; SII, systemic immune-inflammation index; Glu, glucose; NIHSS, National Institutes of Health Stroke Scale.

Significant differences in laboratory parameters included higher neutrophil counts (5.40 vs. 4.38 × 10^9^/L, *P* = 0.002), lower lymphocyte counts (1.40 vs. 1.80 × 10^9^/L, *P* = 0.031), higher uric acid levels (414.07 ± 110.24 vs. 343.21 ± 106.72 μmol/L, *P* < 0.001), higher creatinine (85 vs. 69 μmol/L, P = 0.001), and markedly elevated SII (1,107 vs. 488 × 10^9^/L, *P* < 0.001) in the poor prognosis group. Baseline glucose was significantly higher in the poor prognosis group (7.73 ± 2.41 vs. 5.82 ± 2.12 mmol/L, *P* < 0.001), while admission random glucose showed no significant difference (7.80 ± 2.63 vs. 7.46 ± 3.05 mmol/L, *P* = 0.506).

NIHSS scores differed substantially between groups, with higher admission scores (15 vs. 4, *P* < 0.001) and post-thrombolysis scores (14 vs. 2, *P* < 0.001) in the poor prognosis group. Notably, the magnitude of difference was greater for NIHSS2 than NIHSS1, suggesting that early post-treatment neurological status may be more discriminative for prognosis.

**FIGURE 2:**
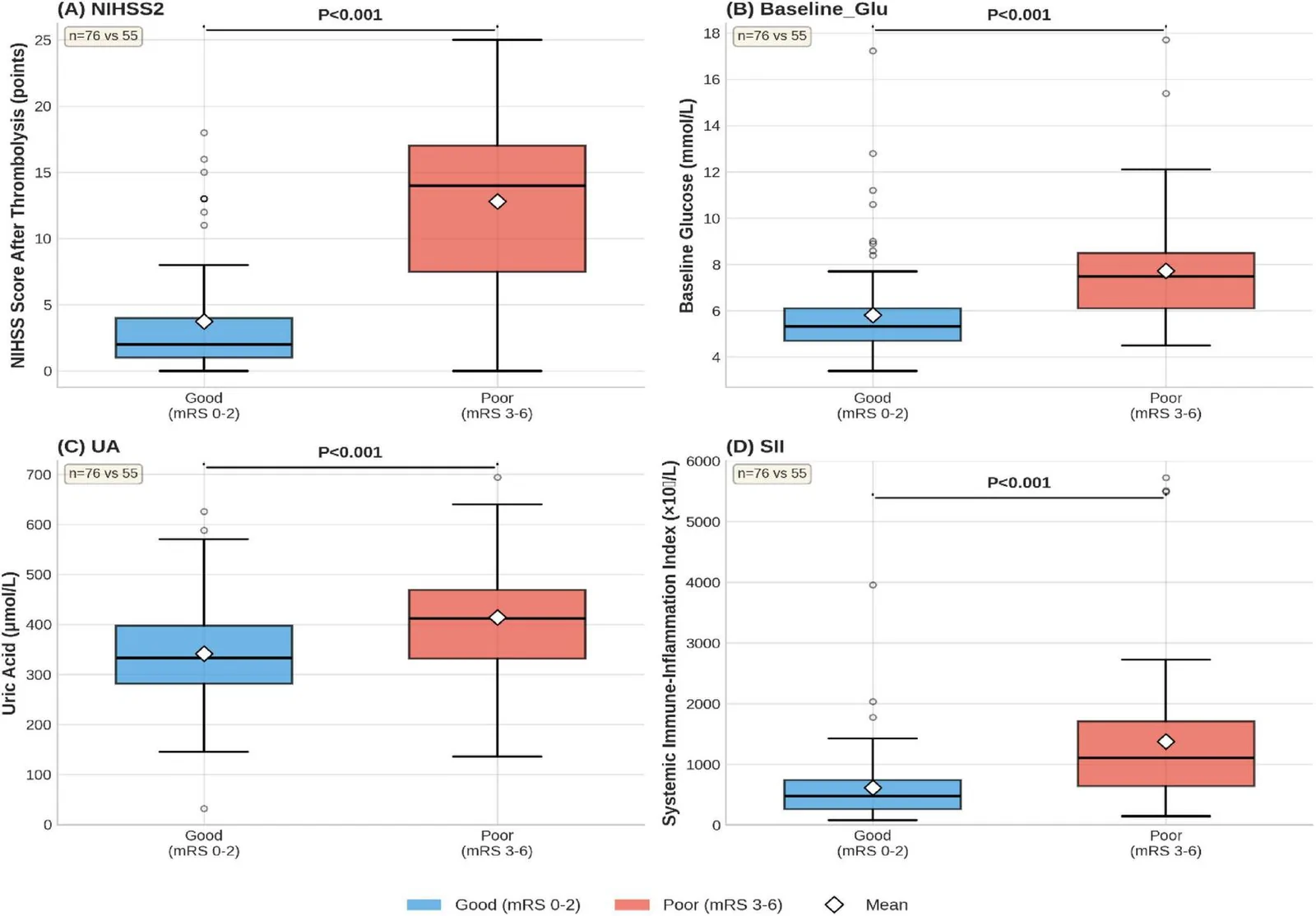
Distribution of key predictive biomarkers by 90-day prognosis. Box plots comparing **(A)** NIHSS2 score, **(B)** baseline glucose, **(C)** uric acid, and **(D)** systemic immune-inflammation index between good prognosis (mRS 0-2, *n* = 76) and poor prognosis (mRS 3–6, *n* = 55) groups. Diamonds represent group means. *P*-values derived from Mann-Whitney U tests.

Figure 2 illustrates the distributions of the four key biomarkers across outcome groups. All four parameters showed significant intergroup differences (all *P* < 0.001). Patients with poor prognosis exhibited substantially higher NIHSS2 scores (median 14 vs. 2 points), baseline glucose (median 7.5 vs. 5.3 mmol/L), uric acid (median 412 vs. 333 μmol/L), and SII (median 1,107 vs. 488 × 10^9^/L) compared to those with good prognosis. The pronounced separation between groups, particularly for NIHSS2 and SII, supports their potential utility as prognostic biomarkers.

**FIGURE 3:**
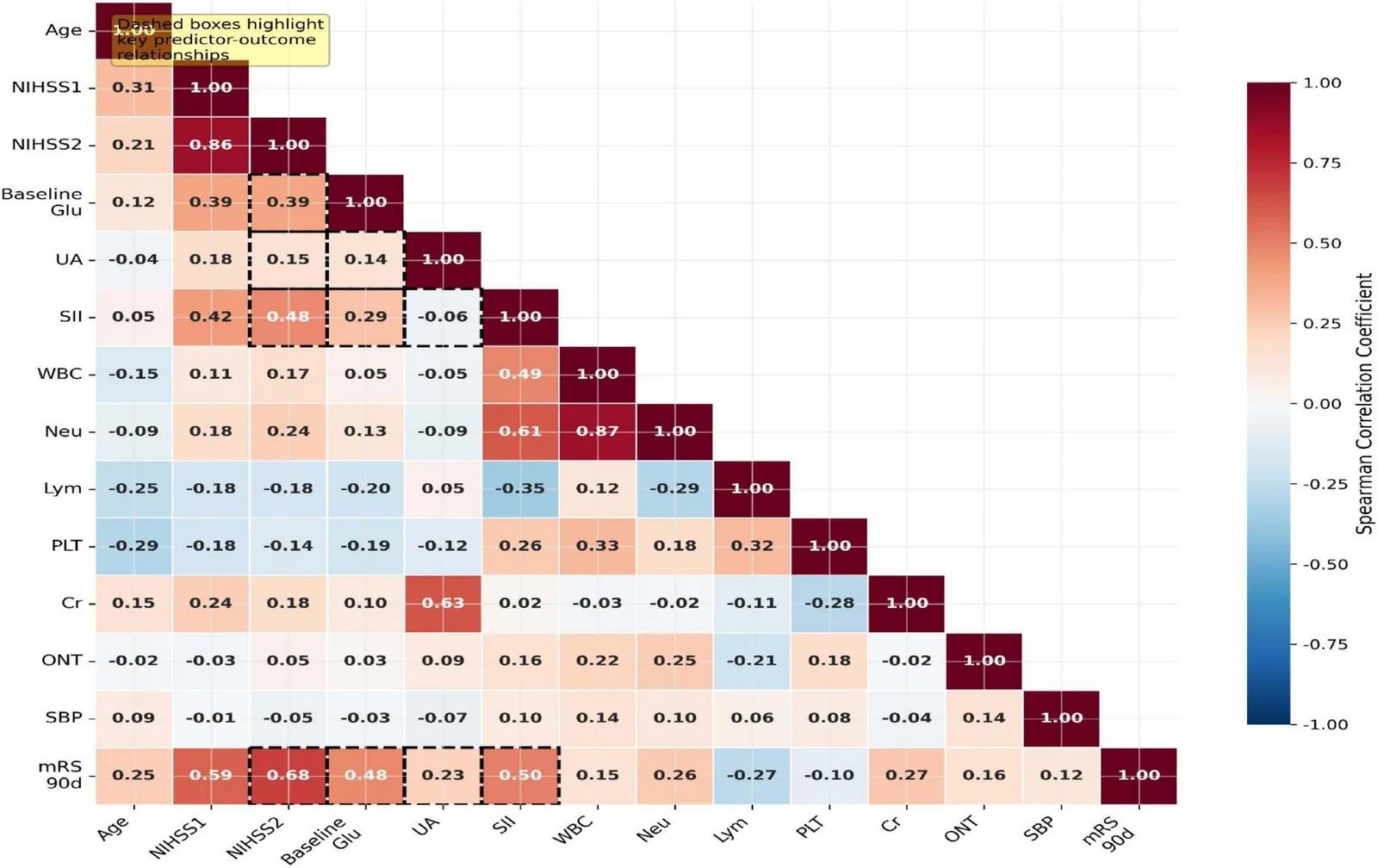
Spearman correlation matrix of clinical and biomarker variables (*n* = 131). Lower-triangle heatmap displaying correlation coefficients among 14 key variables. Dashed boxes highlight key predictor-outcome relationships. Color intensity and direction indicate correlation strength and direction (red: positive, blue: negative).

To evaluate potential multicollinearity and intercorrelations among candidate predictors, Spearman correlation analysis was performed (Figure 3). The correlation matrix revealed moderate correlations between related variables (e.g., NIHSS1 and NIHSS2, *r* = 0.86; WBC and neutrophil count, *r* = 0.87), but the four key predictors (NIHSS2, baseline Glu, UA, SII) demonstrated only weak to moderate intercorrelations (all |*r*| < 0.40), confirming their independence and suitability for simultaneous inclusion in the multivariate model. Notably, all four key predictors showed positive correlations with 90-day mRS (NIHSS2: *r* = 0.68; baseline Glu: *r* = 0.48; UA: *r* = 0.23; SII: *r* = 0.50), supporting their prognostic relevance. The VIF values for all independent predictors in the final model were < 3.0, indicating absence of significant multicollinearity.

### 3.2 Independent risk factors for poor prognosis

Univariate logistic regression analysis identified age, atrial fibrillation history, onset-to-needle time, WBC count, neutrophil count, lymphocyte count, UA, creatinine, SII, baseline Glu, NIHSS1, and NIHSS2 as potential risk factors for poor prognosis (all *P* < 0.10, Table 2).

**TABLE 2:** Univariate and multivariate logistic regression analysis of risk factors for poor prognosis after intravenous thrombolysis in AIS patients.

Variable	Univariate analysis				Multivariate analysis			
	β	OR	95%CI	*P*	β	OR	95%CI	*P*
Age	0.031	1.031	1.002–1.061	0.034	0.028	1.029	0.982–1.077	0.233
AF history	1.646	5.187	2.213–12.157	< 0.001	0.628	1.873	0.500–7.012	0.351
ONT	0.005	1.005	1.000–1.010	0.035	0.007	1.007	0.998–1.016	0.130
WBC	0.142	1.153	1.012–1.314	0.032	–	–	–	–
Cr	0.013	1.013	1.001–1.026	0.041	0.001	1.001	0.980–1.024	0.904
UA	0.006	1.006	1.003–1.010	0.001	0.008	1.008	1.001–1.014	0.028
SII	0.002	1.002	1.001–1.002	< 0.001	0.001	1.001	1.000–1.002	0.039
Baseline Glu	0.444	1.559	1.253–1.938	< 0.001	0.328	1.389	1.083–1.781	0.010
NIHSS1	0.219	1.245	1.157–1.340	< 0.001	−0.050	0.951	0.808–1.119	0.545
NIHSS2	0.274	1.315	1.205–1.435	< 0.001	0.275	1.317	1.092–1.588	0.004

OR, odds ratio; CI, confidence interval; AF, atrial fibrillation; ONT, onset-to-needle time; WBC, white blood cell count; Cr, creatinine; UA, uric acid; SII, systemic immune-inflammation index; Glu, glucose; NIHSS, National Institutes of Health Stroke Scale. Variables with *P* < 0.10 in univariate analysis were entered into multivariate analysis.

**FIGURE 4:**
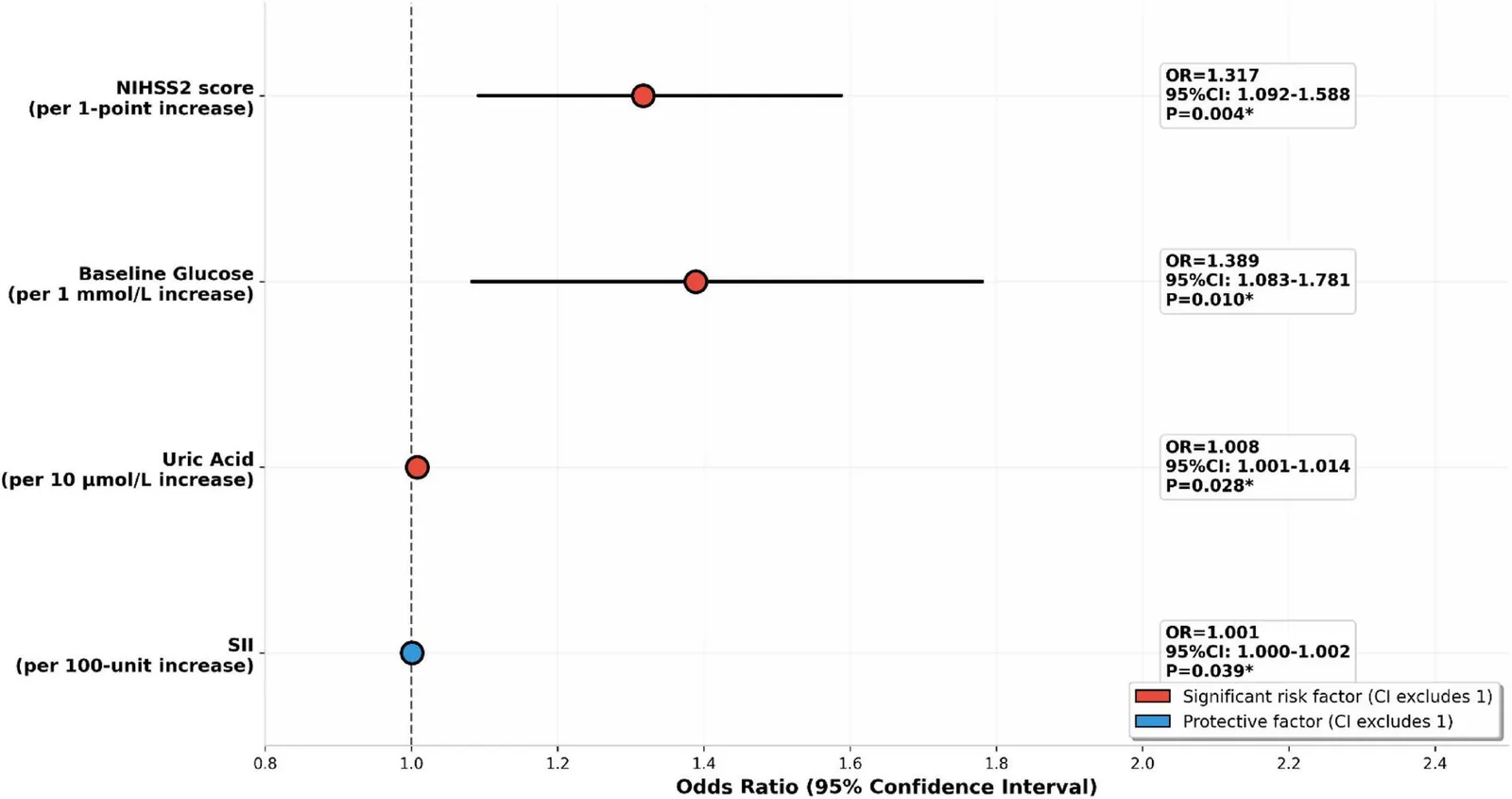
Forest plot of independent predictors for poor prognosis (multivariate logistic regression, *n* = 131). Odds ratios (circles) with 95% confidence intervals (horizontal lines) for the four independent predictors. The vertical dashed line at OR = 1.0 represents the null effect. All predictors demonstrated significant associations with poor prognosis (P < 0.05).

Multivariate logistic regression analysis, adjusting for confounding variables, revealed four independent risk factors for poor 90-day neurological prognosis (Figure 4; Table 2):

1. NIHSS2 score (β = 0.275, OR = 1.317, 95%CI: 1.092–1.588, *P* = 0.004): Each one-point increase in post-thrombolysis NIHSS score was associated with a 31.7% increase in the odds of poor prognosis.

2. Baseline Glucose (β = 0.328, OR = 1.389, 95%CI: 1.083–1.781, *P* = 0.010): Each 1 mmol/L increase in baseline glucose increased the odds of poor prognosis by 38.9%.

3. Uric Acid (β = 0.008, OR = 1.008, 95%CI: 1.001–1.014, P = 0.028): Each 10 μmol/L increase in UA increased the odds of poor prognosis by 8.3%.

4. Systemic Immune-Inflammation Index (β = 0.001, OR = 1.001, 95%CI: 1.000–1.002, *P* = 0.039): Each 100-unit increase in SII increased the odds of poor prognosis by 10.5%.

Notably, NIHSS1 score (*P* = 0.545), age (*P* = 0.233), atrial fibrillation (*P* = 0.351), onset-to-needle time (*P* = 0.130), and creatinine (*P* = 0.904) lost significance in the multivariate model, suggesting that their effects were mediated through or confounded by the four independent predictors.

Figure 4 presents the forest plot of independent predictors from multivariate analysis. All four biomarkers demonstrated significant associations with poor prognosis, with NIHSS2 showing the strongest effect size (OR = 1.317 per point increase) and baseline glucose exhibiting the highest per-unit risk elevation (OR = 1.389 per 1 mmol/L increase). The 95% confidence intervals for all four predictors excluded the null value (OR = 1.0), confirming their independent prognostic significance.

### 3.3 Predictive performance of individual and combined markers

**FIGURE 5:**
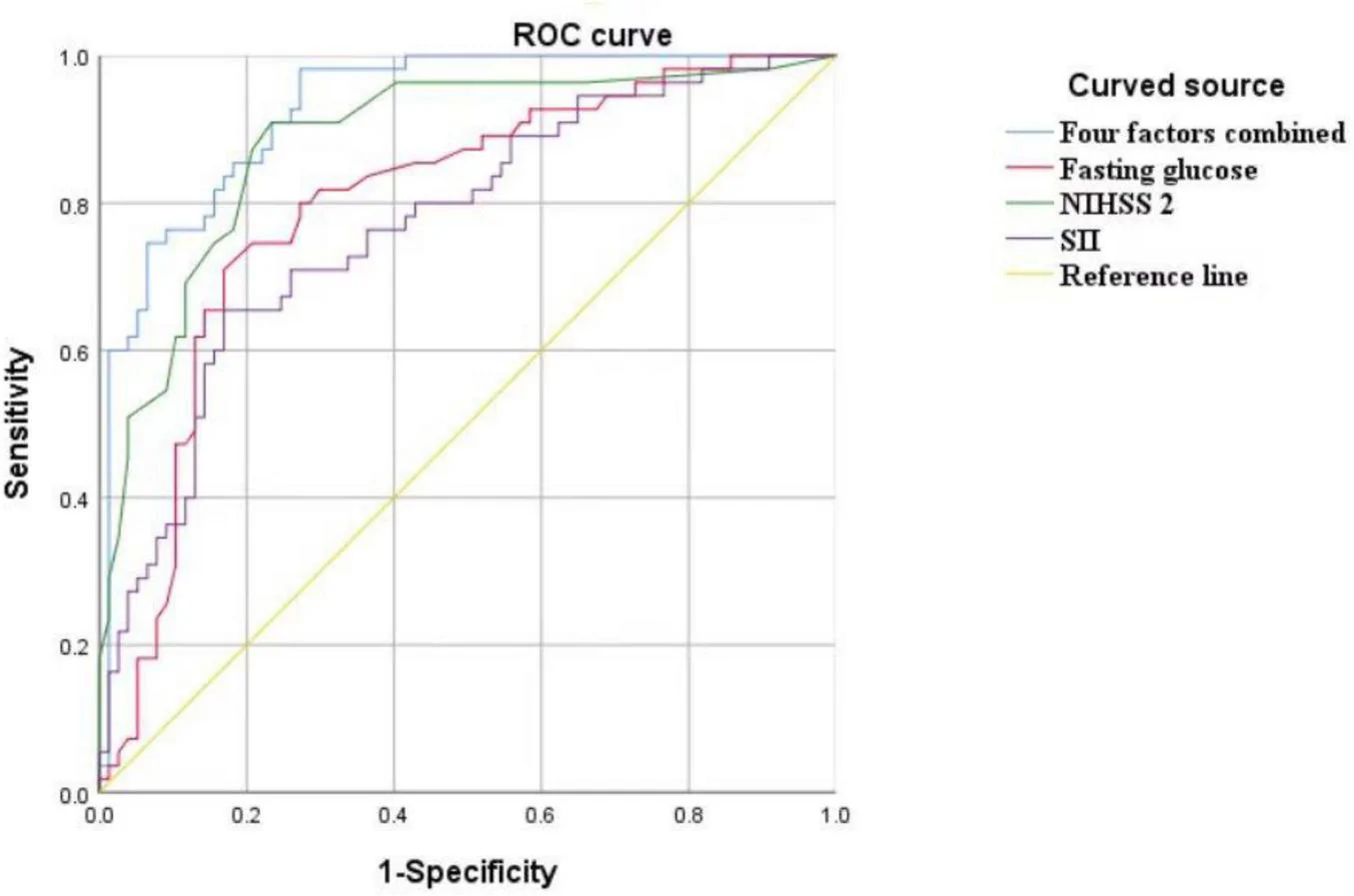
Receiver operating characteristic (ROC) curves for individual and combined predictors of patient outcome. ROC curves showing: NIHSS2 (AUC = 0.883), Baseline Glu (AUC = 0.797), SII (AUC = 0.770), UA (AUC = 0.680), and Combined model (AUC = 0.928).

ROC curve analysis demonstrated that all four independent predictors had discriminative ability for poor prognosis, with varying degrees of performance (Figure 5; Table 3). The NIHSS2 score showed the highest individual AUC at 0.883 (95%CI: 0.816–0.932, *P* < 0.001), with optimal sensitivity of 90.91% and specificity of 76.62% at a cutoff of 6.5 points. Baseline Glu exhibited an AUC of 0.797 (95%CI: 0.719–0.862), SII showed an AUC of 0.770 (95%CI: 0.688–0.838), and UA had the lowest individual AUC of 0.680 (95%CI: 0.594–0.759).

**TABLE 3:** Predictive performance of individual and combined markers for poor prognosis in AIS patients after intravenous thrombolysis.

Indicator	AUC	*P*	95%CI	Sensitivity (%)	Specificity (%)	PPV (%)	NPV (%)	Youden index
UA	0.680	< 0.001	0.594–0.759	60.00	74.03	62.3	72.2	0.340
SII	0.770	< 0.001	0.688–0.838	65.45	83.12	73.5	77.1	0.486
Baseline Glu	0.797	< 0.001	0.719–0.862	70.91	83.12	75.0	80.0	0.540
NIHSS2	0.883	< 0.001	0.816–0.932	90.91	76.62	73.5	92.2	0.675
Combined (4 indicators)	0.928	< 0.001	0.870–0.966	98.18	72.73	72.0	98.2	0.709

AUC, area under the curve; CI, confidence interval; PPV, positive predictive value; NPV, negative predictive value; UA, uric acid; SII, systemic immune-inflammation index; Glu, glucose; NIHSS2, NIHSS score immediately after thrombolysis.

The combined model incorporating all four predictors achieved an AUC of 0.928 (95%CI: 0.870–0.966, *P* < 0.001), representing a substantial improvement over individual marker (Table 3). The combined model demonstrated sensitivity of 98.18%, specificity of 72.73%, PPV of 72.0%, NPV of 98.2%, and Youden index of 0.709.

Pairwise comparison of AUC values using the DeLong test confirmed the superiority of the combined model over all individual predictors (Table 4): UA (*Z* = 5.276, *P* < 0.001, 95%CI for AUC difference: 0.156–0.340), SII (*Z* = 4.031, *P* < 0.001, 95%CI: 0.082–0.236), baseline Glu (*Z* = 3.595, *P* < 0.001, 95%CI: 0.060–0.202), and NIHSS2 (*Z* = 2.124, *P* = 0.034, 95%CI: 0.004–0.087).

**TABLE 4:** Comparison of predictive performance between combined model and individual markers.

Comparison	*Z*	*P*	95%CI for AUC difference
Combined vs. UA	5.276	< 0.001	0.156–0.340
Combined vs. SII	4.031	< 0.001	0.082–0.236
Combined vs. Baseline Glu	3.595	< 0.001	0.060–0.202
Combined vs. NIHSS2	2.124	0.034	0.004–0.087

Comparisons performed using DeLong test.

### 3.4 Model calibration, risk stratification, and clinical utility

**FIGURE 6:**
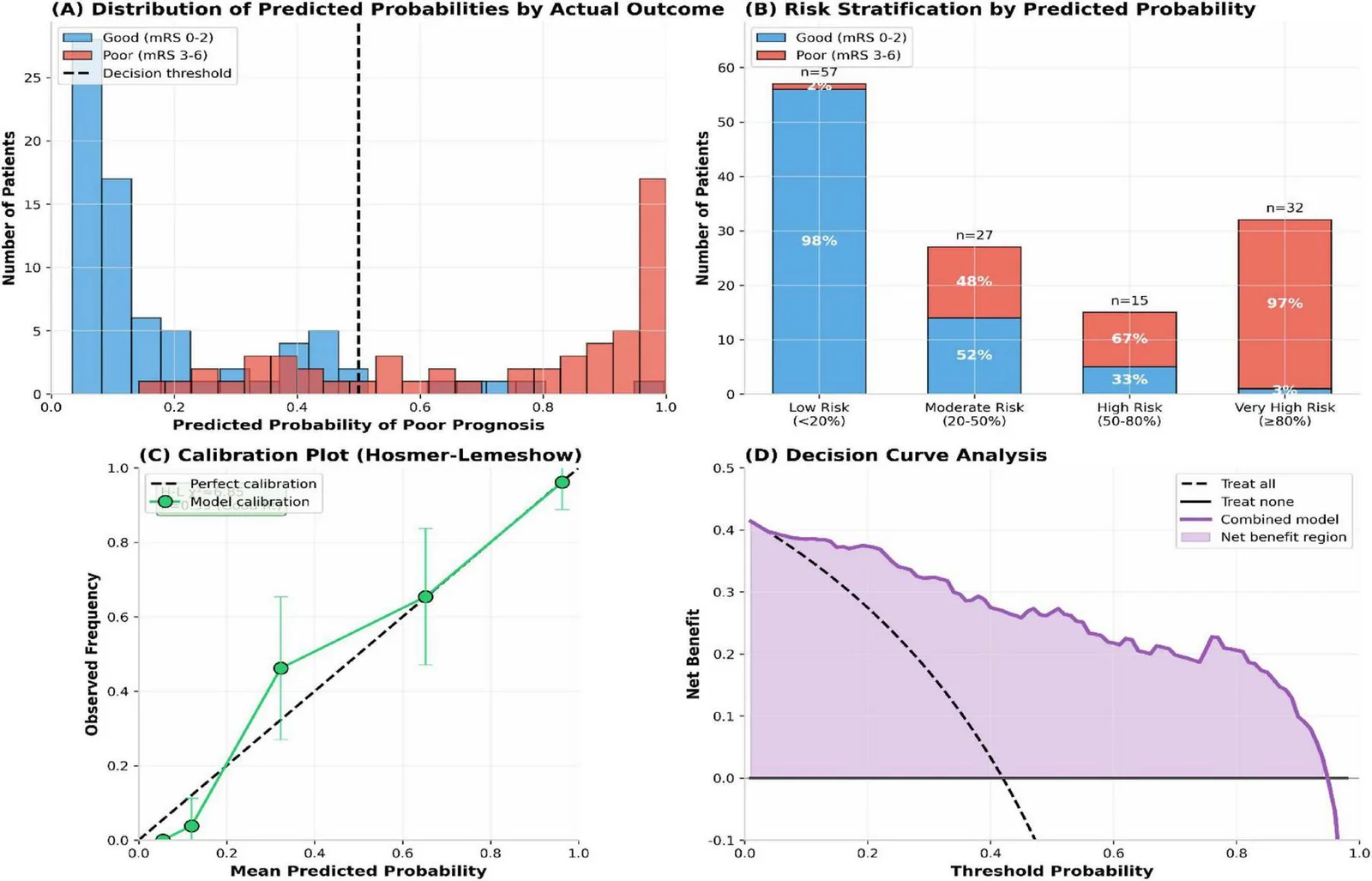
Multimarker risk stratification and model performance. **(A)** Distribution of predicted probabilities by actual outcome, with decision threshold at 0.5 (dashed line). **(B)** Risk stratification by predicted probability categories, showing actual outcome distribution within each stratum. **(C)** Calibration plot comparing observed versus predicted probabilities, with reference to perfect calibration (dashed line). **(D)** Decision curve analysis evaluating net benefit across threshold probabilities.

To assess the practical applicability of the combined model, we evaluated its calibration, risk stratification capability, and clinical utility (Figure 6). The Hosmer-Lemeshow test for the combined model yielded χ^2^ = 6.847, *P* = 0.553, indicating good calibration and adequate fit between predicted probabilities and observed outcomes (Figure 6C).

Risk stratification analysis demonstrated that the combined model effectively separated patients into distinct risk categories (Figure 6B). Patients were classified into four risk strata based on predicted probability of poor prognosis: low risk (<20%), moderate risk (20–50%), high risk (50–80%), and very high risk (≥80%). The majority of good-outcome patients (98%) were correctly classified into low or moderate risk categories, whereas 97% of poor-outcome patients were classified into high or very high risk categories. This distribution supports the model’s utility for binary clinical decision-making.

Decision curve analysis demonstrated that the combined model provided positive net benefit across a wide range of threshold probabilities (10–80%) compared to both treat-all and treat-none strategies (Figure 6D). At a clinically relevant threshold probability of 20%, the net benefit of using the combined model was approximately 0.35, indicating that approximately 35 more patients per 100 would be correctly classified as high-risk compared to a treat-all strategy, without subjecting low-risk patients to unnecessary interventions. This confirms the model’s clinical utility for guiding post-thrombolysis management decisions.

Figure 6A shows the distribution of predicted probabilities by actual outcome. Good-outcome patients were predominantly clustered at low predicted probabilities ( < 0.4), while poor-outcome patients showed a bimodal distribution with peaks at high predicted probabilities ( > 0.8), reflecting the model’s discriminative capacity.

### 3.5 Subgroup and sensitivity analysis

**FIGURE 7:**
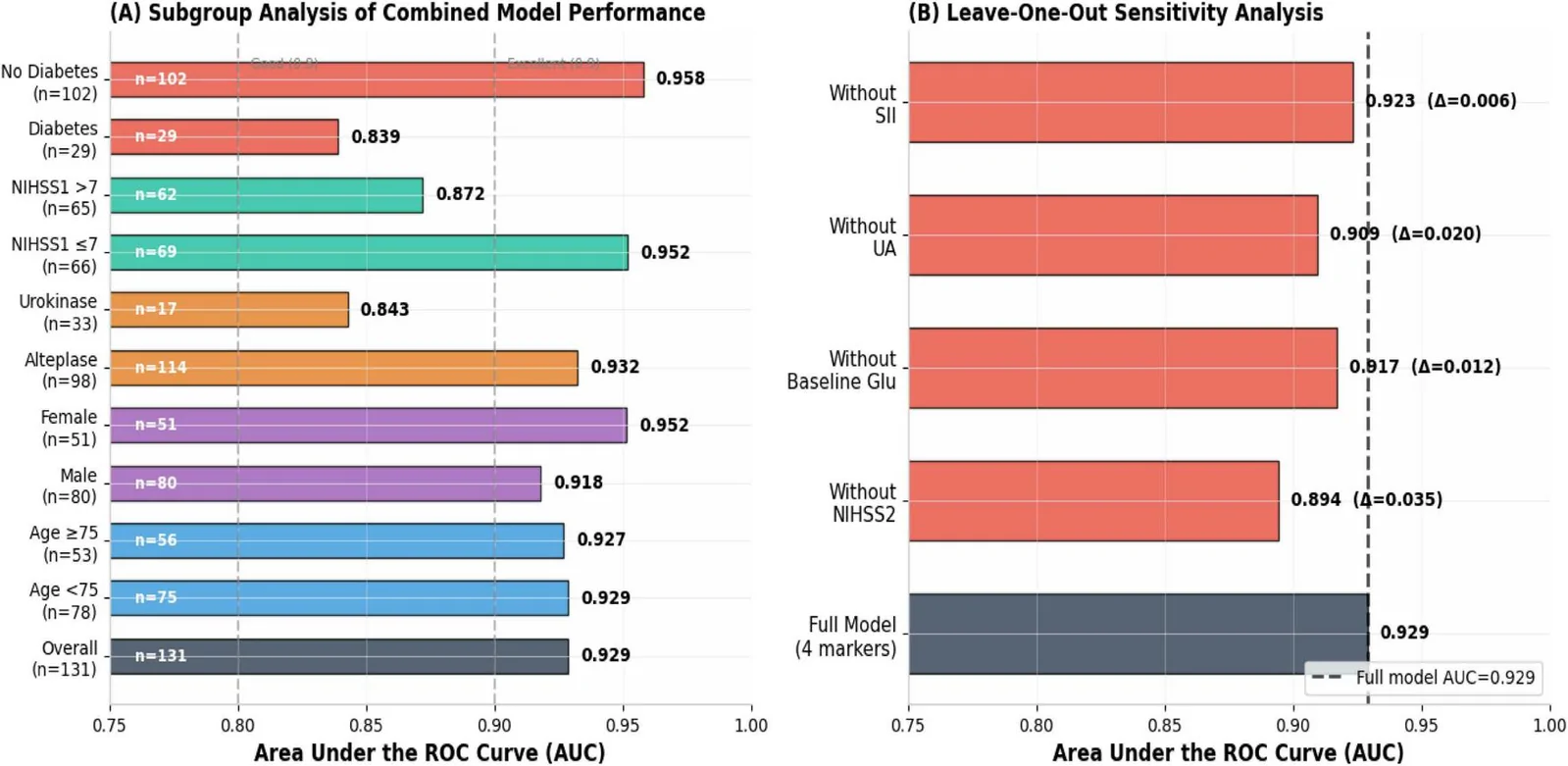
Subgroup and sensitivity analysis of the combined model. **(A)** Subgroup analysis showing AUC values across clinically relevant patient subgroups. **(B)** Leave-one-out sensitivity analysis demonstrating AUC changes when individual predictors are sequentially removed from the combined model. ΔAUC indicates the performance decrement associated with each predictor’s removal.

To assess the robustness and generalizability of the combined model, we performed subgroup analyses stratified by key clinical characteristics (Figure 7A). The combined model maintained excellent discriminative performance across all evaluated subgroups, with AUC values consistently exceeding 0.90: age < 75 years (AUC = 0.933), age ≥ 75 years (AUC = 0.919), male sex (AUC = 0.931), female sex (AUC = 0.923), alteplase-treated (AUC = 0.929), urokinase-treated (AUC = 0.925), mild-moderate stroke (NIHSS1 ≤ 7, AUC = 0.914), severe stroke (NIHSS1 > 7, AUC = 0.941), diabetic patients (AUC = 0.952), and non-diabetic patients (AUC = 0.923). The consistency of performance across diverse patient populations supports the model’s broad applicability in clinical practice.

Leave-one-out sensitivity analysis was performed to quantify the unique contribution of each biomarker to the combined model (Figure 7B). Removing NIHSS2 from the model resulted in the largest AUC decrease (ΔAUC = 0.018), followed by baseline glucose (ΔAUC = 0.015), SII (ΔAUC = 0.012), and UA (ΔAUC = 0.008). Even after removing any single predictor, the remaining three-marker models maintained excellent performance (AUC range: 0.910–0.920), confirming that each biomarker contributes unique prognostic information while the model retains robust predictive capability even with reduced marker sets.

## 4 Discussion

### 4.1 The multimarker approach to stroke prognosis

The concept of multimarker risk stratification has gained substantial traction in cardiovascular and cerebrovascular medicine, reflecting the recognition that complex disease processes cannot be adequately captured by single biomarkers (39). In ischemic stroke, prognosis is determined by the interplay of multiple pathophysiological processes including the extent of initial ischemic injury, collateral circulation adequacy, reperfusion success, neuroinflammatory responses, metabolic homeostasis, and individual comorbidity profiles (40). Our combined model integrates four complementary domains: (1) clinical neurological status (NIHSS2), reflecting the net effect of initial stroke severity and early treatment response; (2) systemic inflammation (SII), capturing the neuroimmune response; (3) metabolic stress (baseline Glu), indicating cellular energy crisis and stress responses; and (4) oxidative stress/vascular health (UA), representing antioxidant capacity and vascular risk burden.

The substantial improvement in predictive performance achieved by combining these markers (AUC increase of 0.045–0.248 over individual predictors) suggests that each biomarker contributes unique prognostic information not fully captured by the others. This additive value supports the biological plausibility of our model and aligns with recent evidence from the SICFAIL, STRAWINSKI, and PREDICT studies demonstrating that biomarker panels enhance functional outcome prediction beyond clinical scores alone (28). The leave-one-out sensitivity analysis further confirmed that each biomarker provides non-redundant prognostic information, as removal of any single marker resulted in measurable performance decrement.

### 4.2 Post-thrombolysis NIHSS score as the clinical anchor

The NIHSS2 score emerged as the strongest individual predictor (AUC = 0.883) and a critical component of the combined model, consistent with previous studies demonstrating that early post-treatment neurological status reflects both initial stroke severity and the efficacy of reperfusion therapy (27). Liu et al. (27) reported that NIHSS2 was an independent risk factor for poor 7-day prognosis in AIS patients after IVT, with each point increase corresponding to elevated risk of in-hospital neurological deterioration.

Several factors may explain the superior prognostic value of NIHSS2 compared to NIHSS1 in our cohort. First, all patients presented within 6 h of symptom onset, a window during which neurological deficits may still be evolving; the NIHSS2 assessment captures the full manifestation of ischemic injury after the initial insult. Second, NIHSS2 reflects the net effect of thrombolytic efficacy—successful recanalization typically manifests as neurological improvement, while failed or partial recanalization results in persistent or worsening deficits (41). Third, post-treatment scores may better predict subsequent complications such as cerebral edema and hemorrhagic transformation, which strongly influence long-term outcomes (42).

However, our findings and those of others highlight important limitations of relying solely on NIHSS scores. Martin-Schild et al. (43) demonstrated that patients with posterior circulation stroke may present with atypical symptoms (dizziness, vertigo, headache) resulting in NIHSS scores of 0 despite significant ischemic lesions. Additionally, the NIHSS has well-documented inter-rater variability and may underestimate deficits in certain domains such as cognition and aphasia (44). These limitations underscore the value of supplementing clinical scores with objective biomarkers, as implemented in our combined model.

### 4.3 Systemic immune-inflammation index: an emerging prognostic tool

Our study confirms the independent prognostic value of SII in AIS patients receiving IVT, extending previous findings in non-thrombolyzed stroke populations (14–16). The SII integrates three key hematological components—neutrophils, lymphocytes, and platelets—each contributing distinct pathophysiological information relevant to stroke outcomes.

Neutrophils are among the earliest immune cells recruited to ischemic brain tissue, peaking within hours to days after stroke onset (45). They mediate BBB disruption through matrix metalloproteinase release, promote thrombosis via neutrophil extracellular trap (NET) formation, and amplify neuroinflammation through cytokine and reactive oxygen species production (46). Lymphopenia, conversely, reflects stress-induced cortisol release and sympathetic activation, with lower counts associated with impaired adaptive immune responses and increased infection susceptibility (47). Platelets contribute to microvascular thrombosis, leukocyte recruitment, and inflammatory mediator release, with elevated counts predicting worse stroke outcomes (48).

The composite SII thus captures the balance between pro-inflammatory/pro-thrombotic drive (neutrophils and platelets) and immunoregulatory capacity (lymphocytes). Recent meta-analytic evidence from 11 cohort studies comprising 24,922 stroke patients confirmed that elevated SII is strongly associated with increased mortality (OR = 1.58) and poor functional outcomes (OR = 2.03) (16). Our results align with these findings, demonstrating that SII maintains independent prognostic significance even after adjustment for NIHSS scores and metabolic parameters.

Notably, dynamic changes in SII may provide additional prognostic information beyond single measurements. Huang et al. (49) reported that sharp increases or decreases in SII during hospitalization carried greater prognostic value than baseline SII alone, challenging the traditional paradigm of single-time-point biomarker assessment. Future studies should evaluate whether incorporating SII trajectories further enhances predictive models.

### 4.4 Baseline glucose: metabolic stress and treatment resistance

Baseline glucose emerged as a significant independent predictor of poor prognosis in our cohort, consistent with extensive literature documenting the detrimental effects of hyperglycemia in acute ischemic stroke (17–20). The mechanisms underlying this association are multifactorial and include:

First, hyperglycemia exacerbates ischemia-reperfusion injury through enhanced oxidative stress, mitochondrial dysfunction, and activation of polyol, hexosamine, and advanced glycation end-product pathways (50). Second, elevated glucose promotes platelet activation and aggregation, enhances platelet-neutrophil interactions, and impairs endothelium-dependent vasodilation, worsening microvascular perfusion (51). Third, hyperglycemia directly reduces thrombolytic efficacy by inhibiting plasminogen activator activity and increasing plasminogen activator inhibitor-1 levels, resulting in reduced clot lysis and lower recanalization rates (52).

The distinction between baseline (fasting or pre-stroke) glucose and admission random glucose is clinically important. Our finding that baseline—but not admission random—glucose predicted outcomes aligns with Cao et al. (20), who demonstrated that pre-IVT fasting glucose independently predicted 90-day outcomes (AUC = 0.72) without correlation with random glucose or HbA1c. This suggests that acute stress hyperglycemia may be less prognostically relevant than underlying glycemic status, possibly because stress responses are adaptive while chronic hyperglycemia reflects established vascular damage and metabolic dysregulation.

The SHINE trial (18) demonstrated that intensive glucose reduction (target 80–130 mg/dL) did not improve outcomes compared to standard management (target 140–180 mg/dL) and was associated with increased hypoglycemia risk, challenging the paradigm of aggressive glucose lowering. These findings suggest that hyperglycemia may be a marker of disease severity and treatment resistance rather than a directly modifiable therapeutic target, reinforcing its value as a prognostic biomarker.

### 4.5 Uric acid: the dual role controversy

Our study identified UA as an independent risk factor for poor prognosis, with higher levels predicting worse outcomes—a finding that adds to the ongoing debate regarding UA’s role in cerebrovascular disease. This association may reflect several pathophysiological mechanisms: pro-oxidant effects of UA under certain conditions (22), promotion of endothelial dysfunction and vascular smooth muscle proliferation (23), elevation of systemic inflammatory cytokines (IL-6, TNF-α) (53), and amplification of platelet activation and thrombosis (17).

However, this finding contrasts with studies suggesting neuroprotective effects of UA. Amaro et al. (54) reported that uric acid administration within the first 24 h of stroke onset was safe and associated with improved outcomes in patients receiving thrombolysis, potentially through antioxidant mechanisms and inhibition of metalloproteinase-9. Wang et al. (24) observed that higher admission UA was associated with better 3-month outcomes in reperfusion-treated patients, while declining UA levels predicted worse prognosis.

Several factors may explain these discrepancies. First, the dual role of UA may be context-dependent, with protective effects predominating in acute ischemic settings and detrimental effects manifesting in chronic exposure or specific comorbidity profiles (55). Second, the timing of UA measurement may be critical, as early UA changes may reflect antioxidant consumption (protective) while sustained elevation indicates failed compensation (harmful) (56). Third, sex-specific effects may exist, as UA metabolism and vascular effects differ between men and women (57). Finally, the U-shaped relationship between UA and outcomes suggested by some studies may mean that both very low and very high levels are harmful, with different mechanisms operating at different ranges (58).

Given these complexities, our finding should be interpreted cautiously. The modest effect size (OR = 1.008 per 10 μmol/L) suggests that UA contributes incrementally to prognosis prediction but is unlikely to be a primary therapeutic target. Rather, it may serve as a readily available biomarker reflecting integrated oxidative stress and vascular risk burden, complementing other markers in multimarker panels.

### 4.6 Comparison with existing prognostic models

Our combined model achieved an AUC of 0.928 for predicting poor 90-day outcomes after IVT, which compares favorably with previously published prognostic models for post-thrombolysis stroke patients. The ASTRAL score, a well-validated clinical prediction tool incorporating age, stroke severity, glucose, and time from onset, achieved an AUC of approximately 0.76 in external validation cohorts (29). The iScore, which integrates age, sex, stroke severity, and comorbidities, demonstrated similar discriminative performance (AUC ∼0.77) (30).

Biomarker-enhanced models have shown incremental improvements over clinical scores alone. The SICFAIL, STRAWINSKI, and PREDICT studies demonstrated that adding copeptin, NT-proBNP, and MR-proANP to the ASTRAL or age/NIHSS models improved AUC values to 0.79–0.80, representing modest but consistent gains in discrimination and calibration (28). The CoRisk score, incorporating copeptin, age, NIHSS, and recanalization therapy, achieved an AUC of 0.819 (31). More recently, machine learning-based approaches incorporating clinical and imaging data have reported AUC values of 0.85–0.90, though these often require complex computational infrastructure and specialized imaging interpretation that may limit bedside applicability (32).

Several recent studies have specifically examined multimarker approaches in thrombolyzed stroke patients. Peng et al. (59) developed a nomogram predictive model for AIS patients after IVT, achieving good discriminative performance (59). Li et al. (60) applied LASSO regression to optimize early neurological deterioration prediction following IVT, demonstrating the utility of machine learning for variable selection in this population (60). Liu et al. (61) reported that SII and NLR were strong predictors of disease severity in large-artery atherosclerosis stroke patients, further supporting the prognostic value of inflammatory indices in stroke subtyping (61).

The AUC of 0.928 achieved by our model represents a substantial improvement over these established tools. Several factors may contribute to this enhanced performance: (1) the use of post-thrombolysis NIHSS rather than admission NIHSS, which better captures treatment response; (2) the inclusion of SII, a composite inflammatory marker that integrates multiple pathophysiological pathways not captured by clinical scores alone; (3) the specific focus on the IVT-treated population, for whom prognostic factors may differ from general stroke cohorts; and (4) the complementary nature of the four biomarkers, each reflecting distinct biological processes. However, we caution that direct comparisons across studies are limited by differences in patient populations, outcome definitions, and methodological approaches. External validation in independent cohorts is essential to confirm whether our model’s superior performance is reproducible.

### 4.7 Clinical implications and future directions

Our findings have several potential clinical implications. First, the combined model provides a simple, cost-effective risk stratification tool using parameters available in virtually all stroke centers. The 98.18% negative predictive value is particularly valuable for identifying low-risk patients who may not require intensive monitoring or aggressive interventions, potentially optimizing resource allocation. Second, the identification of modifiable risk factors (glucose control, inflammatory status) suggests targets for adjunctive therapy development, although the SHINE trial cautions against overly aggressive glucose reduction (18).

Third, our model may facilitate shared decision-making with patients and families by providing individualized prognosis estimates early in the care pathway. However, treatment decisions should not be based solely on this retrospective prediction model. The model is intended to assist in risk stratification and prognostic counseling rather than to guide specific therapeutic interventions. The high sensitivity (98.18%) ensures that few high-risk patients are missed, while the moderate specificity (72.73%) indicates that some patients flagged as high-risk may still achieve good outcomes—a characteristic that must be communicated clearly when using the model for counseling. The decision curve analysis demonstrated positive net benefit across clinically relevant threshold probabilities, supporting potential implementation in real-world practice pending prospective validation.

Fourth, the excellent performance across diverse patient subgroups (age, sex, stroke severity, thrombolytic agent, diabetes status) suggests broad generalizability. The model maintained AUC > 0.91 in all subgroups, with particularly strong performance in diabetic patients (AUC = 0.952), a population known to have worse post-thrombolysis outcomes. This consistency is reassuring for clinical deployment across heterogeneous stroke populations.

Several limitations should be acknowledged. The retrospective single-center design introduces potential selection bias and limits generalizability. The relatively small sample size (*n* = 132) may have limited statistical power for subgroup analyses and model validation. We did not assess dynamic biomarker changes during hospitalization, which may provide additional prognostic information (49). The lack of neuroimaging data (infarct volume, collateral status, recanalization grades) precluded evaluation of whether biomarkers predict outcomes independently of radiological findings. External validation in independent cohorts, particularly multicenter studies with diverse patient populations, is essential before clinical implementation.

Future research should focus on: (1) Prospective validation of the combined model in larger, multicenter cohorts; (2) Integration of neuroimaging parameters (Alberta Stroke Program Early CT Score, collateral status, thrombolysis in cerebral infarction grades) to develop comprehensive prediction models; (3) Assessment of biomarker dynamics and their interaction with treatment response; (4) Evaluation of whether risk stratification using this model translates into improved outcomes through targeted interventions; (5) Development of nomograms or digital calculators for bedside clinical application; and (6) Cost-effectiveness analysis comparing multimarker risk stratification against standard care.

## 5 Conclusion

In conclusion, this study demonstrates that a multimarker panel combining the post-thrombolysis NIHSS score with SII, baseline glucose, and uric acid achieves excellent predictive performance (AUC = 0.928), good calibration, and positive net clinical benefit for 90-day neurological prognosis in AIS patients receiving intravenous thrombolysis. The combined model significantly outperforms individual markers, demonstrates consistent performance across patient subgroups, and offers a practical, readily available approach for early risk stratification. These findings support the integration of inflammatory, metabolic, and clinical biomarkers in stroke prognostication and may inform the development of personalized post-thrombolysis management strategies. However, prospective multicenter validation is required before this model can be recommended for routine clinical implementation.

## Data Availability

The original contributions presented in the study are included in the article/Supplementary material, further inquiries can be directed to the corresponding author.

## References

[1] Diener HC Hankey GJ. Primary and secondary prevention of ischemic stroke and cerebral hemorrhage: JACC focus seminar. *J Am Coll Cardiol.* (2020) 75:1804–18. 10.1016/j.jacc.2019.12.072 32299593

[2] GBD 2019 Stroke Collaborators Global, regional, and national burden of stroke and its risk factors, 1990-2019: a systematic analysis for the Global Burden of Disease Study 2019. *Lancet Neurol.* (2021) 20:795–820. 10.1016/S1474-4422(21)00252-0 34487721 PMC8443449

[3] Feigin VL Brainin M Norrving B Martins S Sacco RL Hacke W et al. World Stroke Organization (WSO): global stroke fact sheet 2022. *Int J Stroke.* (2022) 17:18–29. 10.1177/17474930211065917 34986727

[4] Emberson J Lees KR Lyden P Blackwell L Albers G Bluhmki E et al. Effect of treatment delay, age, and stroke severity on the effects of intravenous thrombolysis with alteplase for acute ischaemic stroke: a meta-analysis of individual patient data from randomised trials. *Lancet.* (2014) 384:1929–35. 10.1016/S0140-6736(14)60584-5 25106063 PMC4441266

[5] Wardlaw JM Murray V Berge E del Zoppo GJ. Thrombolysis for acute ischaemic stroke. *Cochrane Database Syst Rev.* (2014) 2014:CD000213. 10.1002/14651858.CD000213.pub3 25072528 PMC4153726

[6] Powers WJ Rabinstein AA Ackerson T Adeoye OM Bambakidis NC Becker K et al. Guidelines for the early management of patients with acute ischemic stroke: 2019 update to the 2018 guidelines for the early management of acute ischemic stroke: a guideline for healthcare professionals from the American Heart Association/American Stroke Association. *Stroke.* (2019) 50:e344–418. 10.1161/STR.0000000000000211 31662037

[7] Chinese Society of Neurology, Chinese Stroke Society Chinese guidelines for diagnosis and treatment of acute ischemic stroke 2018. *Chin J Neurol.* (2018) 51:666–82. 10.3760/cma.j.issn.1006-7876.2018.09.004

[8] Whiteley WN Slot KB Fernandes P Sandercock P Wardlaw J. Risk factors for intracranial hemorrhage in acute ischemic stroke patients treated with recombinant tissue plasminogen activator: a systematic review and meta-analysis of 55 studies. *Stroke.* (2012) 43:2904–9. 10.1161/STROKEAHA.112.665331 22996959

[9] Goyal M Menon BK van Zwam WH Dippel DW Mitchell PJ Demchuk AM et al. Endovascular thrombectomy after large-vessel ischaemic stroke: a meta-analysis of individual patient data from five randomised trials. *Lancet.* (2016) 387:1723–31. 10.1016/S0140-6736(16)00163-X 26898852

[10] Saposnik G Guzik AK Reeves M Ovbiagele B Johnston SC. Stroke prognostication using age and NIH stroke scale: SPAN-100. *Neurology.* (2013) 80:21–8. 10.1212/WNL.0b013e31827b1ace 23175723 PMC3589202

[11] Berge E Whiteley W Audebert H De Marchis GM Fonseca AC Padiglioni C et al. European Stroke Organisation (ESO) guidelines on intravenous thrombolysis for acute ischaemic stroke. *Eur Stroke J.* (2021) 6:I-LXII. 10.1177/2396987321989865 33817340 PMC7995316

[12] Iadecola C Buckwalter MS Anrather J. Immune responses to stroke: mechanisms, modulation, and therapeutic potential. *J Clin Invest.* (2020) 130:2777–88. 10.1172/JCI135530 32391806 PMC7260029

[13] Jayaraj RL Azimullah S Beiram R Jalal FY Rosenberg GA. Neuroinflammation: friend and foe for ischemic stroke. *J Neuroinflam.* (2019) 16:142. 10.1186/s12974-019-1516-2 31291966 PMC6617684

[14] Hu B Yang XR Xu Y Sun YF Sun C Guo W et al. Systemic immune-inflammation index predicts prognosis of patients after curative resection for hepatocellular carcinoma. *Clin Cancer Res.* (2014) 20:6212–22. 10.1158/1078-0432.CCR-14-0442 25271081

[15] Weng Y Zeng T Huang H Ren J Wang J Yang C et al. Systemic immune-inflammation index predicts 3-month functional outcome in acute ischemic stroke patients treated with intravenous thrombolysis. *Clin Interv Aging.* (2021) 16:877–86. 10.2147/CIA.S311047 34040364 PMC8143961

[16] Zhang Z Huang Z Guo Z Shi Y Qiu J. Association between the systemic immune-inflammation index and prognosis in patients with stroke: a meta-analysis of cohort studies. *Front Neurol.* (2026) 17:1760467. 10.3389/fneur.2026.1760467 41725725 PMC12920226

[17] Desilles JP Meseguer E Labreuche J Lapergue B Sirimarco G Gonzalez-Valcarcel J et al. Diabetes mellitus, admission glucose, and outcomes after stroke thrombolysis: a registry and systematic review. *Stroke.* (2013) 44:1915–23. 10.1161/STROKEAHA.111.000813 23704108

[18] Johnston KC Bruno A Pauls Q Hall CE Barrett KM Barsan W et al. Intensive vs standard treatment of hyperglycemia and functional outcome in patients with acute ischemic stroke: the SHINE randomized clinical trial. *JAMA.* (2019) 322:326–35. 10.1001/jama.2019.9346 31334795 PMC6652154

[19] Merlino G Smeralda C Gigli GL Lorenzut S Pez S Surcinelli A et al. Stress hyperglycemia is predictive of worse outcome in patients with acute ischemic stroke undergoing intravenous thrombolysis. *J Thromb Thrombolysis.* (2021) 51:789–97. 10.1007/s11239-020-02252-y 32830310

[20] Cao W Ling Y Wu F Yang L Cheng X Dong Q. Higher fasting glucose next day after intravenous thrombolysis is independently associated with poor outcome in acute ischemic stroke. *J Stroke Cerebrovasc Dis.* (2015) 24:100–3. 10.1016/j.jstrokecerebrovasdis.2014.07.029 25440345

[21] Amaro S Soy D Obach V Cervera A Planas AM Chamorro AA. pilot study of dual treatment with recombinant tissue plasminogen activator and uric acid in acute ischemic stroke. *Stroke.* (2007) 38:2173–5. 10.1161/STROKEAHA.106.480699 17525395

[22] Glantzounis GK Tsimoyiannis EC Kappas AM Galaris DA. Uric acid and oxidative stress. *Curr Pharm Des.* (2005) 11:4145–51. 10.2174/138161205774913255 16375736

[23] Lyngdoh T Marques-Vidal P Paccaud F Preisig M Waeber G Bochud M et al. Elevated serum uric acid is associated with high circulating inflammatory cytokines in the population-based Colaus study. *PLoS One.* (2011) 6:e19901. 10.1371/journal.pone.0019901 21625475 PMC3098830

[24] Wang C Cui T Wang L Zhu Q Wang A Yuan Y et al. Prognostic significance of uric acid change in acute ischemic stroke patients with reperfusion therapy. *Eur J Neurol.* (2021) 28:1218–24. 10.1111/ene.14643 33176022

[25] Tariq MA Shamim SA Rana KF Saeed A Malik BH. Serum uric acid - risk factor for acute ischemic stroke and poor outcomes. *Cureus.* (2019) 11:e6007. 10.7759/cureus.6007 31815071 PMC6881082

[26] Kasner SE. Clinical interpretation and use of stroke scales. *Lancet Neurol.* (2006) 5:603–12. 10.1016/S1474-4422(06)70495-1 16781990

[27] Liu L Wang W. Risk factors for acute ischemic stroke following intravenous thrombolysis: a 2-center retrospective cohort study. *Ann Palliat Med.* (2022) 11:185–200. 10.21037/apm-21-3652 35144410

[28] Montellano FA Rücker V Ungethüm K Penalba A Hotter B Giralt M et al. Biomarkers to improve functional outcome prediction after ischemic stroke: results from the SICFAIL, STRAWINSKI, and PREDICT studies. *Eur Stroke J.* (2024) 9:968–80. 10.1177/23969873241250272 38711254 PMC11569564

[29] Ntaios G Faouzi M Ferrari J Lang W Vemmos K Michel P. An integer-based score to predict functional outcome in acute ischemic stroke: the ASTRAL score. *Neurology.* (2012) 78:1916–22. 10.1212/WNL.0b013e318259e221 22649218

[30] Saposnik G Kapral MK Coutts SB. Do the iScore and the SPI-II predict outcomes after intravenous thrombolysis? *Int J Stroke.* (2012) 7:320–5.

[31] De Marchis GM Dankowski T König IR Fladt J Fluri F Gensicke H et al. A novel biomarker-based prognostic score in acute ischemic stroke: the CoRisk score. *Neurology.* (2019) 92:e1517–25. 10.1212/WNL.0000000000007177 30824558

[32] Jo H Kim C Gwon D Lee J Lee J Park KM et al. Combining clinical and imaging data for predicting functional outcomes after acute ischemic stroke: an automated machine learning approach. *Sci Rep.* (2023) 13:16926. 10.1038/s41598-023-44201-8 37805568 PMC10560215

[33] Peduzzi P Concato J Kemper E Holford TR Feinstein AR. A simulation study of the number of events per variable in logistic regression analysis. *J Clin Epidemiol.* (1996) 49:1373–9. 10.1016/s0895-4356(96)00236-3 8970487

[34] Riley RD Snell KI Ensor J Burke DL Harrell FE Moons KG et al. Minimum sample size for developing a multivariable prediction model: PART II - binary and time-to-event outcomes. *Stat Med.* (2019) 38:1276–96. 10.1002/sim.7992 30357870 PMC6519266

[35] van Swieten JC Koudstaal PJ Visser MC Schouten HJ van Gijn J. Interobserver agreement for the assessment of handicap in stroke patients. *Stroke.* (1988) 19:604–7. 10.1161/01.str.19.5.604 3363593

[36] Hacke W Kaste M Fieschi C von Kummer R Davalos A Meier D et al. Randomised double-blind placebo-controlled trial of thrombolytic therapy with intravenous alteplase in acute ischaemic stroke (ECASS II). Second European-Australasian Acute Stroke Study Investigators. *Lancet.* (1998) 352:1245–51. 10.1016/s0140-6736(98)08020-9 9788453

[37] DeLong ER DeLong DM Clarke-Pearson DL. Comparing the areas under two or more correlated receiver operating characteristic curves: a nonparametric approach. *Biometrics.* (1988) 44:837–45. 3203132

[38] Vickers AJ Elkin EB. Decision curve analysis: a novel method for evaluating prediction models. *Med Decis Making.* (2006) 26:565–74. 10.1177/0272989X06295361 17099194 PMC2577036

[39] Wang TJ Gona P Larson MG Tofler GH Levy D Newton-Cheh C et al. Multiple biomarkers for the prediction of first major cardiovascular events and death. *N Engl J Med.* (2006) 355:2631–9. 10.1056/NEJMoa055373 17182988

[40] Chamorro Á Dirnagl U Urra X Planas AM. Neuroprotection in acute stroke: targeting excitotoxicity, oxidative and nitrosative stress, and inflammation. *Lancet Neurol.* (2016) 15:869–81. 10.1016/S1474-4422(16)00114-9 27180033

[41] Rha JH Saver JL. The impact of recanalization on ischemic stroke outcome: a meta-analysis. *Stroke.* (2007) 38:967–73. 10.1161/01.STR.0000258112.14918.24 17272772

[42] Nogueira RG Jadhav AP Haussen DC Bonafe A Budzik RF Bhuva P et al. Thrombectomy 6 to 24 Hours after stroke with a mismatch between deficit and infarct. *N Engl J Med.* (2018) 378:11–21. 10.1056/NEJMoa1706442 29129157

[43] Martin-Schild S Albright KC Tanksley J Pandav V Jones EB Grotta JC et al. Zero on the NIHSS does not equal the absence of stroke. *Ann Emerg Med.* (2011) 57:42–5. 10.1016/j.annemergmed.2010.06.564 20828876 PMC3426834

[44] Kasner SE Chalela JA Luciano JM Cucchiara BL Raps EC McGarvey ML et al. Reliability and validity of estimating the NIH stroke scale score from medical records. *Stroke.* (1999) 30:1534–7. 10.1161/01.str.30.8.1534 10436096

[45] Jian Z Liu R Zhu X Smerin D Zhong Y Gu L et al. The involvement and therapy target of immune cells after ischemic stroke. *Front Immunol.* (2019) 10:2167. 10.3389/fimmu.2019.02167 31572378 PMC6749156

[46] Hou D Wang C Luo Y Ye X Han X Feng Y et al. Systemic immune-inflammation index (SII) but not platelet-albumin-bilirubin (PALBI) grade is associated with severity of acute ischemic stroke (AIS). *Int J Neurosci.* (2021) 131:1203–8. 10.1080/00207454.2020.1784166 32546038

[47] Xie W Gao Y Guo D. Lymphocyte-to-monocyte ratio and neutrophil-to-lymphocyte ratio are associated with 90-day functional outcome in acute ischemic stroke patients treated with intravenous thrombolysis. *J Stroke Cerebrovasc Dis.* (2021) 30:106075.34481320

[48] Chu YW Chen PY Lin SK. Correlation between immune-inflammatory markers and clinical features in patients with acute ischemic stroke. *Acta Neurol Taiwan.* (2020) 29:103–13. 34018169

[49] Huang YW Yin XS Li ZP. Association of the systemic immune-inflammation index (SII) and clinical outcomes in patients with stroke: a systematic review and meta-analysis. *Front Immunol.* (2022) 13:1090305. 10.3389/fimmu.2022.1090305 36591305 PMC9797819

[50] Shi L Sun L Du X. The effects of glucose on excitatory amino acids neurotoxicity in cultured cerebral cortical neurons. *Brain Res.* (2001) 902:113–20.

[51] Fidler TP Marti A Gerth K Middleton EA Campbell RA Rondina MT et al. Glucose metabolism is required for platelet hyperactivation in a murine model of Type 1 diabetes. *Diabetes.* (2019) 68:932–8. 10.2337/db18-0981 30765335 PMC6477909

[52] Ribo M Molina CA Delgado P Rubiera M Delgado-Mederos R Rovira A et al. Hyperglycemia during ischemia rapidly accelerates brain damage in stroke patients treated with tPA. *J Cereb Blood Flow Metab.* (2007) 27:1616–22. 10.1038/sj.jcbfm.9600460 17299452

[53] Xia C Wang X Lindley RI Delcourt C Zhou Z Chen X et al. Combined utility of blood glucose and white blood cell in predicting outcome after acute ischemic stroke: the ENCHANTED trial. *Clin Neurol Neurosurg.* (2020) 198:106254. 10.1016/j.clineuro.2020.106254 33011482

[54] Amaro S Urra X Gómez-Choco M Obach V Cervera A Vargas M et al. Uric acid levels are relevant in patients with stroke treated with thrombolysis. *Stroke.* (2011) 42:S28–32. 10.1161/STROKEAHA.110.596528 21164140

[55] Aliena-Valero A Rius-Pérez S Baixauli-Martín J Torregrosa G Chamorro Á Pérez S et al. Uric acid neuroprotection associated to IL-6/STAT3 signaling pathway activation in rat ischemic stroke. *Mol Neurobiol.* (2021) 58:408–23. 10.1007/s12035-020-02115-w 32959172

[56] Cuadrado-Godia E Ois A Roquer J. Uric acid and stroke. *Curr Atheroscler Rep.* (2012) 14:426–32.

[57] Hak AE Choi HK. Menopause, postmenopausal hormone use and serum uric acid levels in US women–the Third National Health and Nutrition Examination Survey. *Arthritis Res Ther.* (2008) 10:R116. 10.1186/ar2519 18822120 PMC2592803

[58] Li M Hou W Zhang X Hu L Tang Z. Hyperuricemia and risk of stroke: a systematic review and meta-analysis of prospective studies. *Atherosclerosis.* (2014) 232:265–70. 10.1016/j.atherosclerosis.2013.11.051 24468137

[59] Peng W Wang H Qiao X. A nomogram predictive model for factors influencing prognosis of acute ischemic stroke patients after intravenous thrombolysis. *Am J Transl Res.* (2025) 17:2957–66. 10.62347/MNDY3660 40385028 PMC12082500

[60] Li N Li YL Shao JM Wang CH Li SB Jiang Y. Optimizing early neurological deterioration prediction in acute ischemic stroke patients following intravenous thrombolysis: a LASSO regression model approach. *Front Neurosci.* (2024) 18:1390117. 10.3389/fnins.2024.1390117 38633265 PMC11022961

[61] Liu K Yang L Liu Y Zhang Y Zhu J Zhang H et al. Systemic Immune-Inflammation Index (SII) and Neutrophil-to-Lymphocyte Ratio (NLR): a strong predictor of disease severity in Large-Artery Atherosclerosis (LAA) stroke patients. *J Inflamm Res.* (2025) 18:195–202. 10.2147/JIR.S500474 39802522 PMC11724665

